# The Origin of Skin Dendritic Cell Network and Its Role in Psoriasis

**DOI:** 10.3390/ijms19010042

**Published:** 2017-12-23

**Authors:** Tae-Gyun Kim, Sung Hee Kim, Min-Geol Lee

**Affiliations:** 1 Department of Dermatology, Cutaneous Biology Research Institute, Severance Hospital, Yonsei University College of Medicine, Seoul 03722 Korea; tgmed83@yuhs.ac (T.-G.K.); SYPZWSH@yuhs.ac (S.H.K.); 2 Brain Korea 21 PLUS Project for Medical Science, Yonsei University College of Medicine, Seoul 03722, Korea

**Keywords:** dendritic cells, ontogeny, psoriasis, skin

## Abstract

Dendritic cells (DCs) are heterogeneous groups of innate immune cells, which orchestrate immune responses by presenting antigens to cognate T cells and stimulating other types of immune cells. Although the term ‘DCs’ generally represent highly mixed subsets with functional heterogeneity, the classical definition of DCs usually denotes conventional DCs (cDCs). Skin contains a unique DC network mainly composed of embryo precursor-derived epidermal Langerhans cells (LCs) and bone marrow-derived dermal cDCs, which can be further classified into type 1 (cDC1) and type 2 (cDC2) subsets. Psoriasis is a chronic inflammatory skin disease, which is principally mediated by IL-23/IL-17 cytokine axis. In the psoriatic skins, DCs are prominent cellular sources for TNF-α and IL-23, and the use of blocking antibodies against TNF-α and IL-23 leads to a significant clinical improvement in psoriatic patients. Recent elegant human and mouse studies have shown that inflammation-induced inflammatory DCs, LCs, dermal cDC2, and monocyte-derived DCs are pivotal DC subsets in psoriatic inflammation. Thus, targeting specific pathogenic DC subsets would be a potential strategy for alleviating and preventing DC-derived IL-23-dependent psoriatic inflammation and other inflammatory dermatoses in the future.

## 1. Introduction

Our body is constantly exposed to a variety of external antigens and allergens in daily life. Most of those antigenic challenges are generally sub-immunogenic and can be effectively controlled by a defined immunological tolerance and negative regulatory mechanisms equipped in our immune system [1]. Our immune system should distinguish self-antigens from foreign ones to prevent catastrophic immunity, and those unwanted exaggerated immune responses result in diverse allergic, autoimmune, and immune-mediated inflammatory diseases [2]. Skin is a specialized integumentary organ having a cornified outer layer stratum corneum which principally provides a physical protection against the diverse external stimuli [3]. In addition, intriguingly, skin harbors a large number of immune cells which are mainly comprised of T cells, innate lymphocytes (NK cells, NKT cells, and innate lymphoid cells), mast cells, and antigen-presenting cells, which evidently symbolizes that skin is also important as a primary immune sentinel of our body [4]. However, hyperactive immune responses due to a complex interaction between genetic and epigenetic causes may trigger chronic inflammatory skin disorders, such as atopic dermatitis and psoriasis.

Among the skin-resident immune cells, there is a distinct population of dendritic cells (DCs), which are characterized by distinct dendritic morphology and the surface expression of a high level of integrin α X (CD11c) and major histocompatibility complex class II molecules (MHC II) [5]. Functionally, DCs are professional antigen-presenting cells which uptake and process protein antigens to present them to antigen-specific T lymphocytes. Cutaneous DCs continuously patrol the interface between skin and outer environments, and transmigrate to the regional draining lymph nodes (LNs) where DCs interact with the highly enriched number of T cells [6]. Although LN migration of antigen-bearing DCs efficiently elicits productive T cell immunity, in certain instances, migratory DCs also actively dampen T cell immune responses via currently not well understood cellular and molecular mechanisms [7]. In addition, mature DCs produce high amounts of pro-inflammatory cytokines which specifically direct T cell differentiation programs and other immune cell activation [8]. In this regard, one can easily imagine that a balance between activation and deactivation of DCs is a crucial mechanism for regulating optimal degree of immune responses. However, chronically-inflamed skins, such as lesional atopic dermatitis and psoriasis contain a highly increased number of cytokine-producing mature DCs which are believed to drive chronic T cell activation and disease-specific immune responses [9]. Those specific subsets of DCs play highly inflammatory roles in the disease pathogenesis, which enables us to define that they are pathogenic DCs. In this review, we discussed our current knowledge of the skin DC network and its pathogenic role in the inflammatory skin disorders, especially focusing on psoriasis. Understanding of the cutaneous DC network both in the health and disease may provide opportunities to develop novel strategies to alleviate and prevent chronic inflammatory diseases of the skin through modulating cutaneous DCs.

## 2. Origins of the Skin Dendritic Cell Network

**Figure 1:**
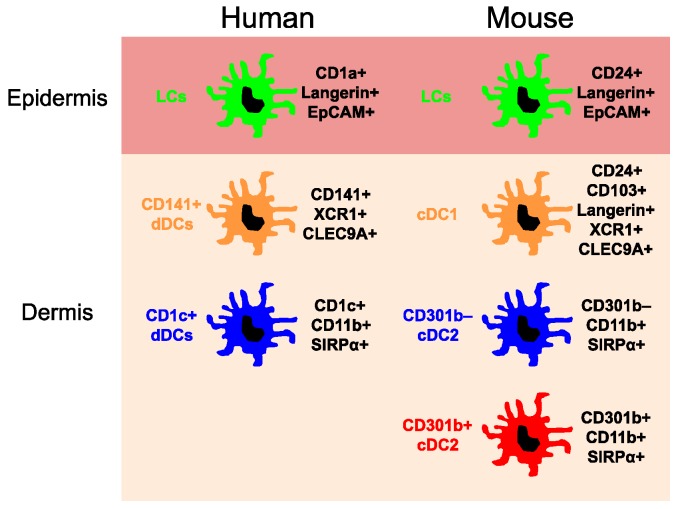
Major dendritic cell subsets in the human and mouse skin. CLEC9A, C-type lectin domain containing 9A; EpCAM, Epithelial cell adhesion molecule; SIRPα, Signal regulatory protein α; XCR1, Chemokine (C motif) receptor 1.

Skin is a multi-layered barrier organ. The outer layer epidermis is primarily composed of particular epithelial cell keratinocytes, which undergo a vertical differentiation process and ultimately become a cornified cell layer (stratum corneum). The lower layer dermis consists of heterogeneous connective tissues, such as collagens and vascular structures. The location of cutaneous DCs is largely distributed to the epidermis and upper dermis which can be visualized by the immunohistological staining of skin with anti-MHC II antibody and three-dimensional leukocyte mapping of the skin [10]. Under steady-state conditions, according to the developmental origins, transcription factor dependency, and surface marker expression patterns, currently at least three major DC subsets have been well-described; (1) epidermal Langerhans cells (LCs); (2) dermal type 1 conventional DCs (cDC1); and (3) dermal type 2 conventional DCs (cDC2) (Figure 1). Under inflammatory conditions, however, additional subtypes of DCs arise in the inflamed skin, such as plasmacytoid DCs, inflammatory myeloid DCs, and monocyte-derived DCs [11]. Although the term ‘DCs’ generally represent highly-mixed cell populations, classical definition of DCs in vivo usually represent conventional DCs (cDCs) which arise from the DC-committed bone marrow progenitors in response to a specific hematopoietin, FMS-like tyrosine kinase 3 ligand (FLT3L) [12].

### 2.1. Epidermal Langerhans Cells

LCs, as the sole DC subset, reside in the quiescent epidermis. LCs were initially discovered from the human skin by Paul Langerhans in 1868. Through the gold chloride staining technique, he described LCs as intraepidermal receptors for extracutaneous signals of the nervous system [13]. Nearly 100 years later, the identity of LCs was changed to immune cells since they expressed MHC II molecules, Fc receptors, and C3 complement receptors [14,15,16,17]. In line with their expression of MHC II molecules, LCs were subsequently shown to present antigens to T cells and become more potent T cell stimulators upon two to three days of in vitro culture [18,19,20,21,22,23]. These cardinal features of LCs positioned them as model subsets of migratory tissue DCs, in which uptake peripheral antigens and migrate to local draining lymph nodes where they initiate strong T cell immune responses. Early studies from allogeneic skin graft and bone marrow transplantation experiments to identify the origin of LCs had shown that LCs were derived from the circulating bone marrow progenitors [24,25]. However, LCs were capable of proliferating within the epidermis in situ [26] and an elegant murine parabiosis study showed that LCs maintained their cell number via self-renewal throughout life under steady-state conditions without any bone marrow precursor input [27]. Studies searching for the endogenous LC precursors demonstrated that primitive LC precursors prenatally infiltrated the epidermis during the late stage of embryo development where they underwent burst proliferation and differentiation process during the first week of newborn mice [28]. A subsequent in vivo lineage tracing study revealed that LC precursors originated from the two discrete embryonic myeloid progenitors, namely yolk sac-derived macrophages and fetal liver monocytes [29]. In adult mice, the epidermal LC network was sustained by actively dividing progenitor-like LCs and their daughter cells, which formed proliferative LC units revealed by fate-mapping experiments [30]. Certain inflammatory stimuli could enhance LC migration and local proliferation, which partially explain how the adult LC pool recovers after LCs leave the epidermis [28]. However, upon severe inflammation, such as ultraviolet irradiation, chemical exposure, and mechanical perturbation, Gr-1^hi^ circulating monocytes are rapidly recruited to the skin and transformed to mature LCs [27,31]. In this step, compartmentalized hair follicle keratinocytes produced a different set of chemokines, including CCL2, CCL20, and CCL8, to finely regulate the trafficking of LC precursors around the hair follicles where they entered the epidermis [32]. 

Those monocyte-derived LCs, which rapidly populated the inflamed epidermis, were inhibitors of DNA binding 2 (Id2)-independent and lived a relatively short period in the epidermis (short-term LCs). Soon after, Id2-dependent long-lived LCs emerged and repopulated the whole epidermis (long-term LCs) [33]. Although the origins of Id2-dependent long-lived LCs are not yet clear, studies have suggested that bone marrow-derived precursors of unknown origin may play a role in this process [32,33,34]. In contrast to cDCs in the dermis, which are dependent on FLT3 receptor signaling for the development and homeostasis, LC development specifically requires colony stimulating factor 1 (CSF1) receptor signaling [31]. In the skin, keratinocyte-derived IL-34 is a ligand for CSF1 receptor and IL-34 knockout mice showed defects in the development, homeostasis, and maintenance of LCs [35,36]. In addition, development and maintenance of the LC network critically depends on transforming growth factor beta (TGF-β) signaling and both autocrine and paracrine sources for TGF-β1 were involved [37,38,39]. Interestingly, TGF-β1 was not only crucial for the LC network formation, but also regulated spontaneous LC migration out of the epidermis, as inducible depletion of TGF-β receptor signaling on LCs led to an accelerated LC migration in vivo [40]. TGF-β1 activity in the skin was closely regulated by integrins expressed by keratinocytes including α_v_β_6_ integrin in the interfollicular epidermis and α_v_β_8_ integrin in the follicular epidermis [41]. LC development is dependent on specific transcription factors, such as Id2, Runt-related transcription factor 3 (Runx3), and PU.1 [42,43,44] and mTOR signaling pathway [34,45]. Compared to the transcription factor dependency, it is currently less well understood how LC development and homeostasis is regulated by epigenetic controls. Dicer-dependent microRNAs have been implicated in the maintenance of LC quantity [46]. Our group has recently demonstrated that homeostatic maintenance of the LC network is critically regulated by one genome tailor protein, CCCTC-binding factor (CTCF), in vivo [47]. Using a conditional gene knockout mouse system, we found that, although the neonatal LC network formation was not affected, CTCF-deficient LCs showed a reduced homeostatic proliferation in the adult mouse epidermis [47]. These results implicate that the maintenance of epidermal LC homeostasis is finely regulated by the epigenetic regulatory machineries which may induce a core gene expression signature for LCs.

Human epidermal LCs are characterized by the bright expression of CD1a and CD207 (Langerin). Although the developmental origin of human LCs has not been well understood, LC precursors colonized the embryonic epidermis and differentiated into the mature LCs similar to the murine LC development [48]. It has been shown that the culture system of human CD34+ hematopoietic stem cells with multiple hematopoietic cytokines supplemented with TGF-β could mimic epidermal LC differentiation [49]. By using this in vitro model system, AXL Receptor Tyrosine Kinase (AXL) and Bone Morphogenetic Protein 7 (BMP7) have been implicated in human epidermal LC differentiation [50,51]. However, recent studies have revealed that circulating CD1c+ cDC2 could differentiate into LC-like DCs in vitro, indicating that the origin and homeostasis of human LCs would be different from those of mice [52,53]. The differences between human and murine LC biology should be carefully evaluated in the future.

### 2.2. Dermal Conventional Dendritic Cells

cDCs represent classical myeloid DCs commonly derived from FLT3L-dependent DC-committed BM progenitors. cDCs are found in both lymphoid and non-lymphoid tissues, including the dermis of the skin. Dermal cDCs mainly locate in the upper area of the dermal skin [10]. Bone marrow-derived cDC-restricted progenitors, pre-cDCs, terminally differentiate into mature cDCs in the skin [12]. Those pre-cDCs were not able to produce monocytes or macrophages, which established a distinct DC lineage in vivo [54,55,56]. As already discussed, based on the differential surface marker expression, transcription factor dependency, and functions, cDCs are divided into two discrete population, cDC1 and cDC2 [57]. A single-cell resolution transcriptomic approach has shown that the commitment of DC progenitors to either cDC1 or cDC2 subsets was determined at the pre-cDCs stage [58]. Comparative biology analysis between mouse and human DC subset has provided deep insights for understanding of the ontogeny and function of DC subsets across the species [59]. In humans, there is a circulating pre-cDC population found in cord blood and bone marrow, as seen in mice [60]. Dermal cDC1 lineage expresses surface markers, including CD24, CD103, CD207, and XCR1 in mice, and CD141 (BDCA-3) and XCR1 in humans. Dermal cDC2 lineage is characterized by expressing CD11b and CD172α (SIRPα) in mice, and CD1c (BDCA-1), CD11b, and CD172α in humans [61,62]. In the murine skin, there is another minor FLT3L-responsive cDC population which is devoid of expressing surface markers for cDC1 and cDC2, and whose development depends on the Kruppel-like factor 4 (KLF4) transcription factor [63,64]. In human skin, there is an additional CD14+ DC subset which is considered as monocyte-derived cells different from self-perpetuating tissue-resident macrophages [65].

Pre-cDCs and all types of cDCs are characterized by the shared expression of transcription factor zinc finger and BTB domain containing 46 (Zbtb46), although Zbtb46 was dispensable for the cDC development [66,67]. Development of cDC1 was dependent on transcription factor interferon regulatory factor 8 (Irf8) and basic leucine zipper ATF-like transcription factor 3 (Batf3). Irf8-deficient mice showed a reduced number of cDC1 [68] and DC-specific *Irf8* knockout experiments revealed that Irf8 was a terminal selector of the cDC1 lineage [69]. cDC1 development was also abrogated in *Batf3* knockout mice [70] and, importantly, Batf3 promoted autoactivation of *Irf8* gene expression, which maintained the cDC1 lineage [71]. Compared to dermal cDC1, the transcription factor requirement for dermal cDC2 development is less well understood because of a highly heterogeneous nature of CD11b+ myeloid lineage cells found in the skin [72]. Although dermal cDC2 specifically expresses interferon regulatory factor 4 (Irf4) transcription factor, Irf4 was not involved in dermal cDC2 development [73,74,75]. Rather, Irf4 was critical for the migration or survival of migratory dermal cDC2 in the draining lymph nodes and priming T cell responses. Several studies have shown that CD301b was a valuable surface marker which distinguished a certain DC subset from the non-lymphoid tissues, including skin [76,77,78]. Our group has recently demonstrated that the murine CD301b+ dermal DC subset was a skin-specific subpopulation of FLT3 signaling-dependent dermal cDC2, which was not observed in the secondary lymphoid organ, the spleen [79]. Interestingly, both in vitro and in vivo development of CD301b+ cDC2 were dependent on granulocyte macrophage-colony stimulating factor (GM-CSF) [79], which has long been implicated in the development of monocyte-derived inflammatory DCs [80]. Recent elegant mouse genetic studies have revisited the functional role for GM-CSF in the control of cDC homeostasis since the lack of GM-CSF signaling led to a significantly reduced cell number of cDC1 and cDC2 in the skin [81]. Thus, emerging evidence suggests that both FLT3L and GM-CSF play a concerted action for the development of the dermal skin DC network in murine skin. However, the physiological role for GM-CSF in the human dermal DC network formation and homeostasis remains to be determined.

## 3. Dendritic Cells in the Pathogenesis of Human Psoriasis

Psoriasis is a chronic inflammatory skin disorder characterized by erythematous and scaly plaques with epidermal hyperplasia. Although psoriasis was considered as a disease of the hyper-proliferation of aberrant keratinocytes, a very large body of genetic and immunological studies has emphasized that psoriasis is an immune-mediated disease [82]. Gene expression profiles of the lesional psoriasis have established that psoriasis is mainly induced by IL-23 and type 17 (IL-17A, IL-17F, and IL-22) cytokines [83]. Psoriasis frequently develops on the damaged skin (Koebner phenomenon), which indicates that innate danger signals may trigger psoriatic inflammation. Xenograft of the unaffected skins of the psoriatic patients onto the immune-deficient mice led to an auto-induction of psoriatic lesions, indicating an importance of resident immune cells and local immune environments [84]. In this model, plasmacytoid DCs (pDCs), which produce a large amount of type I interferon in response to TLR7 and TLR9 ligation, were rapidly recruited and played an important role during the initiation phase of the psoriatic plaque formation [85]. pDC recruitment was correlated with a distinct expression of chemerin by dermal fibroblasts and endothelial cells, which induced chemerin receptor ChemR23+ pDC chemotaxis [86]. Self-DNA released by damaged skin and antimicrobial peptide LL-37 could form self-DNA-LL-37 complex, which directly activated pDCs to produce type I interferon to promote functional maturation of myeloid DCs in psoriasis [87,88]. In the psoriatic lesions, one can find a dramatic increase in the number of dermal myeloid DC populations and, interestingly, those infiltrating DCs showed CD1c− phenotype and expressed proinflammatory molecules TNF-α and iNOS [89,90]. Psoriatic inflammatory DCs were capable of polarizing and stimulating Th1/Th17 T cells, and psoriatic lesions contained an increased number of Th1/Th17 cell population [90,91]. Because of the pro-inflammatory features of the psoriatic myeloid DCs, they are considered as an ‘inflammatory type’ of DCs arising during the skin inflammation [9]. The identity of the psoriatic inflammatory DCs is yet poorly understood, however, there was a report to show that Slan+ DCs were IL-23-producing inflammatory DCs in psoriasis [92]. However, transcriptome analysis of the psoriatic dermal inflammatory DCs revealed that gene expression profiles of psoriatic CD1c− DCs were most close to those of CD1c+ dermal cDC2, suggesting that psoriatic inflammatory DCs might originate from dermal cDC2 under the inflammatory conditions [93]. Apart from dermal inflammatory DCs, recent studies have demonstrated an emergence of epidermal inflammatory DCs in the psoriatic epidermis, which also produced IL-23 and IL-1β similar to dermal inflammatory DCs [94]. In addition, it has been shown that CD5 surface marker-expressing LCs and dermal DCs were more potent in stimulating T cell proliferation and cytokine production, however, they already existed in the healthy skin [95]. Additional studies will definitely be needed to elucidate the underlying nature of psoriatic inflammatory epidermal and dermal DCs.

**Figure 2:**
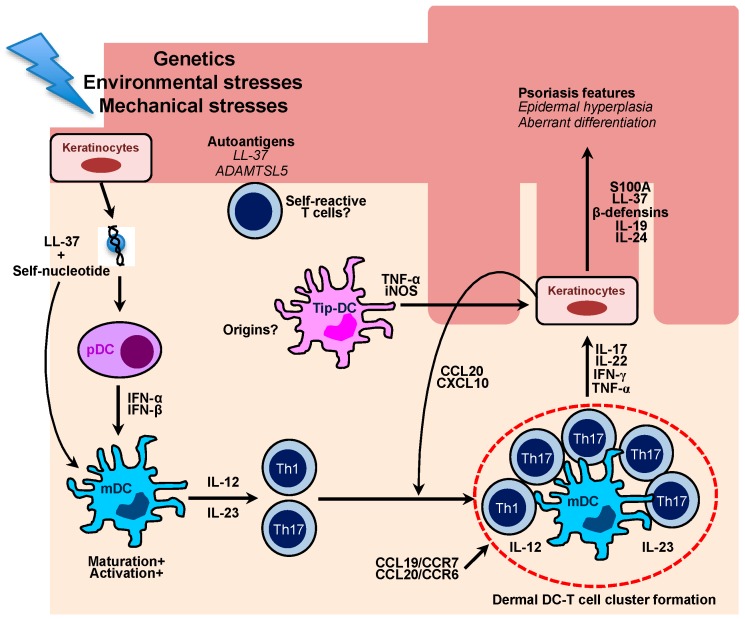
Schematic diagram for the role of multiple cutaneous DCs in the pathogenesis of human psoriasis. Damaged keratinocytes release self-nucleotide, which forms LL-37-self-nucleotides complexes. The complexes directly stimulate plasmacytoid DCs to produce a large amount of type I interferons, which leads to maturation and activation of myeloid DCs. Activated DCs are able to produce IL-12 and IL-23 which primes and stimulates Th1 and Th17 cells, respectively. In the psoriatic lesions, there are cellular aggregates, which mainly comprise skin-infiltrating mDCs and Th1/Th17 cells. The formation of DC-T cell clusters is associated with CCL19/CCR7 and CCL20/CCR6 chemokine axis, which ultimately drives chronic T cell activation in situ. Effector cytokines mainly produced by T cells induce keratinocyte proliferation and aberrant differentiation, which are key characteristics of psoriasis. Cytokine-stimulated ketatinocytes also secrete chemokines, such as CCL20 and CXCL10, which efficiently recruit Th17 and Th1 cells into the lesions. Distinct inflammatory type of DCs, namely Tip-DCs arise in the psoriatic lesions and produce a large amount of pro-inflammatory cytokine to potentiate psoriatic inflammation. Recent studies have highlighted an autoimmune nature of psoriasis as psoriatic patients harbor self-reactive T cells clones against putative psoriasis autoantigens, including LL-37 and A disintegrin-like and metalloprotease domain containing thrombospondin type 1 motif-like 5 (ADAMTSL5).

Psoriatic inflammatory DCs participate in the psoriatic inflammation mainly through producing key pathogenic cytokines, including TNF-α and IL-23. The use of TNF-α blockers in human psoriasis led to a clinical improvement with a reduced IL-17 molecular signature of the lesions [96]. In addition, blocking of IL-23 by IL-12/23p40 or IL-23p19 blocker results in highly effective clinical outcomes compared to conventional immunosuppressive agents, further implicating that lesional IL-23 production from inflammatory DCs is a key molecular event in the psoriasis pathogenesis [83]. Psoriatic DCs also closely localize with the lesional T cells, likely through certain chemokine-chemokine receptor interactions, such as the CCL20/CCR6 system, which may explain a continuous activation of pathogenic T cells in the psoriatic skins [97,98]. Thus, targeting the culprit chemokine system for DC/T cell cluster formation in psoriasis could be a novel therapeutic modality in the future (Figure 2).

## 4. Dendritic Cells in the Pathogenesis of Murine Experimental Psoriasis

Although there are no animal models that precisely mimic human psoriasis, topical application of TLR7 agonist imiquimod (IMQ) has been extensively used for murine experimental model for psoriasis [99]. Topical treatment of IMQ results in several features of psoriasis-like skin inflammation, such as erythema, thickening, and scale, which are immunologically dependent on IL-23 and IL-17 axes, as seen in human psoriasis [100,101]. It has been shown that DC-depleted mice were significantly protected from IMQ-induced inflammation and, importantly, had a reduced number of lesional IL-17A-producing T lymphocytes, indicating that DCs played a central role in this psoriatic mouse model [102]. DCs mediated IMQ-induced psoriatic inflammation through the DC-intrinsic MyD88-dependent toll-like receptor signaling pathway [103]. However, as already discussed in the previous section, cutaneous DCs are quite heterogeneous and the cellular nature of the inflammatory psoriatic DCs is still elusive. Thus, elucidating cutaneous DC subsets, which drive psoriatic inflammation in the IMQ model could provide a novel insight to understand the pathogenesis. The role for epidermal LCs is somewhat controversial as there have been conflicting results from experiments using LC-depleting mice [103,104,105]. However, LCs are likely to stimulate IL-17-producing CD1a-responsive, auto-reactive T cells through presenting lipid antigens, which might contribute to the psoriasis pathogenesis [106]. Identification of IL-23-producing DC subsets would be an important approach to define the pathogenic inflammatory DCs in psoriasis. One study showed that Langerin marker negative dermal DCs, which mainly denote dermal cDC2, were capable of producing IL-23 in response to IMQ treatment [103]. In this regard, our group examined whether CD301b+ cDC2, which is a discrete subpopulation of dermal cDC2, was involved in the IMQ-induced psoriasis-like inflammation. Indeed, depletion of CD301b+ dermal cDC2 resulted in less severe psoriatic inflammation compared to wild-type mice, and CD301b+ dermal cDC2 subset produced a high level of IL-23p19 in the lesional psoriatic skins [79]. These results indicate that CD301b+ dermal cDC2 is a critical cellular player mediating an early phase of developing psoriasis. Hence, targeting human analogue of CD301b+ dermal cDC2 may be a promising strategy to alleviate or prevent human psoriasis. However, currently human counterparts for murine CD301b+ dermal cDC2 have not been investigated yet. Recent comparative biology approaches combined with single cell sequencing technologies will definitely shed light on this issue in the near future [107]. One study also emphasized the role for monocyte-derived DCs in psoriatic inflammation, which needs to be further tested in human psoriasis and psoriasis xenograft models in the future [108]. Furthermore, efforts to clarify the possible association between human inflammatory DCs and murine monocyte-derived DCs will be required to link the DC-centered pathogenesis of psoriatic inflammation between human and mouse systems.

## 5. Conclusions

In this review, we discussed our current knowledge of the cutaneous DC network both in human and mice, and its implication in the pathogenesis of psoriasis. Recent elegant comparative biology studies have revealed the shared ontogenetic properties among human and mouse skin DC subsets, which enabled us to more deeply understand the pathogenic role of individual DC subset in psoriatic inflammation. As DCs are essential to initiating T cell immune responses, the development of novel strategies for targeting specific DC subsets could bring clinical benefit of long-term disease control in psoriasis.

## Abbreviations

ADAMTSL5	A disintegrin-like and metalloprotease domain containing thrombospondin type 1 motif-like 5
AXL	AXL receptor tyrosine kinase
Batf3	Basic leucine zipper ATF-like transcription factor 3
BM	Bone marrow
BMP7	Bone morphogenetic protein 7
cDCs	Conventional dendritic cells
CSF1	Colony stimulating factor 1
DCs	Dendritic cells
FLT3L	FMS-like tyrosine kinase 3 ligand
GM-CSF	Granulocyte macrophage-colony stimulating factor
Id2	Inhibitor of DNA binding 2
Irf	Interferon regulatory factor
KLF4	Kruppel-like factor 4
LCs	Langerhans cells
RUNX3	Runt-related transcription factor 3
TGF-β	Transforming growth factor beta
Zbtb46	Zinc finger and BTB domain containing 46
